# Clinical exigencies, psychosocial realities: negotiating HIV pre‐exposure prophylaxis beyond the cascade among gay, bisexual and other men who have sex with men in Canada

**DOI:** 10.1002/jia2.25211

**Published:** 2018-11-26

**Authors:** Peter A Newman, Adrian Guta, Ashley Lacombe‐Duncan, Suchon Tepjan

**Affiliations:** ^1^ Factor‐Inwentash Faculty of Social Work University of Toronto Toronto ON Canada; ^2^ School of Social Work University of Windsor Windsor ON Canada; ^3^ School of Social Work University of Michigan Ann Arbor MI USA

**Keywords:** HIV prevention, PrEP, risk behaviour, sexual health, social stigma, qualitative research

## Abstract

**Introduction:**

Notwithstanding the efficacy of oral pre‐exposure prophylaxis (PrEP) in clinical trials, a number of obstacles exist to achieving population‐level impact among gay, bisexual and other men who have sex with men (GBM). However, few studies have explored the subjective experiences of GBM PrEP users and non‐users in the community, outside of clinical trials. The objectives of this study were to explore GBM's experiences of considering, accessing and using (or not using) PrEP, and to understand emerging sexual health, social and community issues among GBM in the PrEP era.

**Methods:**

From October 2015 to March 2016, we purposively sampled PrEP‐naïve and PrEP‐experienced GBM from community organizations and health centres in Toronto, Canada. In‐depth, 45‐ to 90‐minute semi‐structured interviews explored PrEP perspectives and decision‐making, access, initiation, use over time, sexual practices and psychosocial considerations. Interviews were recorded, transcribed verbatim, uploaded into NVIVO, reviewed using thematic analysis and then contrasted with the PrEP cascade.

**Results:**

Participants included PrEP users (n = 15) and non‐users (n = 14) (mean age = 36.7 years; SD = 8.2), largely gay‐identified (86.2%), cisgender male (89.7%) and white (79.3%). Themes indicate not only correspondences, but also limitations of the PrEP cascade by complicating a user/non‐user binary and challenging the unilateral presupposition that HIV risk perception leads to PrEP acceptance. Findings further call into question assumptions of a linear stage progression and retention in care as a universal endpoint, instead revealing alternate trajectories of seasonal or intermittent PrEP use and, for some, an end goal of terminating PrEP. GBM's narratives also revealed potent psychological/affective experiences of untethering sex from HIV anxiety; multifaceted PrEP stigma; and challenges to sexual norms and practices that complicate existing behavioural prevention strategies and sexual and social relationships.

**Conclusions:**

An expanded PrEP cascade should consider alternate trajectories of use based on dynamic relationships and behavioural risks that may call for seasonal or intermittent use; systemic barriers in access to and sustaining PrEP; and multiple end goals including PrEP maintenance and discontinuation. Incorporating GBM's lived experiences, evolving preferences, and psychosocial and community‐level challenges into PrEP implementation models, rather than a circumscribed biomedical approach, may more effectively support HIV prevention and GBM's broader sexual and psychological health.

## 1 Introduction

Notwithstanding the efficacy of oral pre‐exposure prophylaxis (PrEP) 1 and its approval in the US 2, Canada and other jurisdictions 3, a number of obstacles exist to achieving population‐level impact among gay, bisexual and other men who have sex with men (GBM) 4, 5, 6, 7, 8. In response to these challenges, several models of a PrEP “cascade” or continuum of care have been developed 7, 9, 10 that outline major steps in implementation, from health systems (e.g. identification of high‐risk GBM, screening PrEP candidates, linkage to care, etc.) and individual perspectives (e.g. perception of HIV risk, awareness of PrEP, access to healthcare, etc).

A number of quantitative studies have identified gaps across the cascade: missed opportunities in translating PrEP interest into initiation 5, racial disparities in initiation 11, 12 and drop‐offs (or “failures”) at each stage 9, 10, 13. Quantitative metrics have also been applied to assess individual's PrEP knowledge, awareness, willingness and intentions to use PrEP 14, 15, 16, 17, 18, and correlates of uptake 5, with extensive attention to potential “risk compensation” 2, 7, 19, 20.

Reflective of a general shift to an HIV prevention paradigm dominated by biomedical approaches 21, 22, 23, few studies have explored the subjective experiences of GBM in navigating PrEP. Several qualitative studies have explored perspectives of “PrEP‐naïve” GBM 24, 25, 26, 27. Qualitative investigations with PrEP‐experienced GBM have largely been conducted in the context of clinical trials or demonstration projects 19, 28, 29, 30, 31, 32, 33, 34, with supports for uptake (e.g. no‐cost PrEP, assistance navigating insurance coverage) and adherence (e.g. financial incentives, counselling), among GBM who may be characterized as early adopters. Scant qualitative research has been conducted with GBM PrEP users and non‐users in community settings 35. Understanding real‐world experiences of PrEP, including the meanings with which it is imbued, its impact on sexual practices, relationships and GBM communities, may have significant implications for its clinical deployment in a “PrEP cascade” – and its impact in the real world beyond clinical trials 21, 22, 36, 37.

The objectives of this study were (1) to explore, in depth, the experiences of GBM in considering, accessing and using (or not using) PrEP; and (2) to understand emerging sexual health, social and community issues among GBM in the PrEP era.

## 2 Methods

### 2.1 Study design and sample

From October 2015 to March 2016, we conducted an exploratory qualitative study to understand the experiences of PrEP users and non‐users in Toronto, Canada. We used purposive sampling based on participants’ self‐identifying as GBM, and as PrEP users or non‐users who had thought about PrEP. Recruitment was conducted by posting flyers in community venues serving GBM and through word‐of‐mouth.

### 2.2 Data collection

Data were collected using a brief, self‐administered socio‐demographic questionnaire and in‐depth semi‐structured 45‐ to 90‐minute interviews. Questionnaire items included demographics and insurance status (i.e. coverage for PrEP). Face‐to‐face interviews explored sexual health and relationships, PrEP knowledge, access and decision‐making; and additionally, for PrEP users, experiences with initiation, engagement with care, taking PrEP and long‐term goals; and for PrEP non‐users, anticipated comfort accessing PrEP and sexual practices. Interviews were conducted (by AG) at a private office at the University of Toronto or a mutually agreed upon public location.

### 2.3 Data analysis

We used Fisher's exact tests to assess demographic differences between PrEP users and non‐users. Interviews were audio‐recorded, transcribed verbatim, uploaded into NVIVO (NVIVO: QSR International Pty Ltd, version 10.0, Burlington, MA, USA) and coded using thematic analysis 38, 39. Each transcript was coded independently by two investigators (AG and ALD). After reading and re‐reading the transcripts for familiarization, we inductively generated initial codes through a process of open coding in which we tagged segments of text that represented underlying themes. ALD and AG met biweekly during the coding process to compare codes and discuss emerging themes. A third coder (ST) then conducted secondary coding of select transcripts. Finally, ALD, AG and PAN reviewed the themes and determined major themes that emerged from the data. Differences in coding and themes were resolved by consensus. Rigour was established through memoing (i.e. reflective note‐taking), negative case‐finding and creation of an audit trail (i.e. to document research activities and decisions made in the analytic process) 40, 41. Lastly, we interfaced themes with the PrEP cascade to identify correspondences, dissonances, and psychological/affective and social phenomena that may impact on PrEP implementation.

### 2.4 Ethical considerations

The study was approved by the Research Ethics Board at the University of Toronto. All participants provided written informed consent and received a $30 honorarium.

## 3 Results

### 3.1 Participant demographics

Participants’ (n = 29) mean age was 36.7 years (SD = 8.2). Most of them self‐identified as gay (n = 25; 86.2%), cisgender male (n = 26; 89.7%) and white (n = 23; 79.3%). The majority had some college education or above (n = 24; 82.8%) and were employed full‐time (n = 18; 62.1%). About half had insurance that covered PrEP (n = 15, 51.7%). By design, participants were equally divided between PrEP users (n = 15; 51.7%) and non‐users (n = 14; 48.3%). PrEP users were significantly more likely to identify as gay versus bisexual/queer and to have insurance that covers PrEP (see Table 1).

**Table 1:** Participant characteristics among gay, bisexual and other men who have sex with men (N = 29) in Toronto, Canada, October 2015 to March 2016

Variable	n	%
Age (mean, SD; years)	36.7	±8.2
Gender		
Cisgender man	26	89.7
Trans man	3	10.3
Sexual orientation^a^		
Gay	25	86.2
Bisexual/queer/pansexual	4	13.8
Ethnicity		
White	23	79.3
Person of colour	6	20.7
Education		
≤High school	5	17.2
≥Some college	24	82.8
Employment		
Full‐time	18	62.1
Not full‐time	11	37.9
Insurance covers PrEP^a^		
No/do not know	14	48.3
Yes	15	51.7

a Pre‐exposure prophylaxis (PrEP) users were significantly more likely to identify as gay (*p* = 0.042, Fisher's exact test) and to have insurance that covered PrEP (*p* = 0.0001, Fisher's exact test) compared to PrEP non‐users.

Major themes, dimensions and exemplar quotations are presented below and in Appendix 1. Quotations from participants are labelled with a participant number, PrEP user or non‐user and age.

### 3.2 HIV risk behaviours

Regardless of PrEP use, participants generally described having multiple partners, using condoms inconsistently and employing a range of strategies for managing sexual risk, including condom use with particular (e.g. non‐primary) partners, serosorting and seropositioning. A participant explained, “I like ‘natural’; I hate condoms” (P9, non‐user, 40 years). However, some participants who opted against PrEP reported consistent condom use and no negative feelings about condoms: “Yeah, I use condoms religiously” (P25, non‐user, 29 years).

### 3.3 HIV risk awareness

In the context of pervasive HIV risk behaviours, participants described sexual anxiety that resulted in cyclical engagement in sexual risk practices and healthcare: condomless sex, anxiety and HIV testing to confirm their (negative) status, sometimes multiple times per year: “I don't like that anxiety; you have sex with someone and then you don't know if they're positive or what, and then you have to go get tested” (P19, PrEP user, 48 years).

Some participants who had not initiated PrEP use described HIV risk behaviours and the use of testing, but demonstrated low perceived risk and no anxiety about acquiring HIV: “No, and I'll tell you why [I don't use condoms]. I am one of those true tops. I test every year, if not twice a year; I'm negative” (P16, non‐user, 47 years). Others described eschewing PrEP based on sexually transmitted infection (STI) risk awareness: “It's not some magic pill that prevents you from contracting any sort of STI” (P25, non‐user, 29 years).

Some participants, largely non‐users, described concerns that PrEP use would lead them to increase sexual risk‐taking: “I'd probably be taking a much more laissez‐faire attitude towards safety” (P5, non‐user, 47 years); “I saw it as almost like a Russian roulette approach to having unprotected, risky sex…” (P13, non‐user, 38 years). Thus, sexual risk practices and associated HIV and STI risk awareness were construed by some GBM as a rationale for *not* using PrEP.

### 3.4 PrEP information seeking

All PrEP users and some non‐users reported seeking out information in scientific (e.g. academic journals) and/or community‐based sources (AIDS service organization websites). Some did this very intentionally: “I read every single study…every single clinical practice guideline that had been published” (P10, PrEP user, 27 years). Others indicated valuing discussion of PrEP and exploring experiences of PrEP users: “Yes, I did look it up online, but I prefer talking to someone rather than seeing many things online. I read blogs of people on PrEP” (P18, PrEP user, 31 years).

### 3.5 PrEP access

Lack of insurance coverage for PrEP emerged as a significant concern among non‐users: “…one of the biggest reasons why I am not on PrEP is because I don't think I'd be able to afford it” (P4, non‐user, 24 years). Another participant described his partial coverage as a barrier: “It would actually be difficult for me to access therapies because I only have 80% drug coverage” (P1, non‐user, 33 years).

Some PrEP‐naïve participants indicated uncertainty about their insurance coverage.

### 3.6 Linkage to PrEP care

Most participants who sought out PrEP did not report barriers in linkage to care nor discriminatory reactions from healthcare providers. PrEP access was facilitated by an urban environment with many LGBTQ‐friendly services and physicians, many of whom also provide care to people living with HIV. As participants explained, “I actually sat down and had a long discussion with my doctor who is here in Toronto and he's been working on HIV for decades” (P14, PrEP user, 42 years). Another PrEP user recounted, “So, I saw him [my doctor], and told him about this. And he just said, ‘so, okay, you want to go on PrEP?’ ‘Yes.’ That's it!” (P12, PrEP user, 42 years).

### 3.7 Prescribed PrEP

Participants generally described a smooth process of procuring a prescription for PrEP: “…when I eventually did go in, it was really straightforward…conversation about why I wanted to take it” (P20, PrEP user, 30 years). Participants also recounted positive experiences in pharmacies, which tended to be in the downtown core, considered LGBTQ‐friendly, and knowledgeable about HIV: “The pharmacist that I see is well‐versed in PrEP…” (P10, PrEP user, 27 years).

### 3.8 PrEP initiation

PrEP initiation was described with a range of actions, from taking PrEP immediately, to taking a PrEP selfie, to waiting. Participants also articulated a range of emotions: feeling happy, proud, overwhelmed or ambivalent. A participant noted, “I finally got my ducks in a row to take it, so it felt pretty good” (P22, PrEP user, 23 years). Another participant described ambivalence, regret and delayed initiation due to no longer considering himself to be at risk for HIV infection:

I kind of convinced myself and took it but then I regretted it… And I kept it in the drawer. Actually, on the very first day I thought that I would start using it tomorrow, and I thought maybe next week, maybe the week after. And I think it's been like two weeks now and I haven't used it because…thinking I stopped hooking up with guys (P29, PrEP user, 40 years).

Several narratives of PrEP initiation indicated the use of social media, including engagement in a PrEP group on Facebook or taking a “PrEP selfie” for online or private use: “I took a picture; I took a selfie, just like hundreds of other people have done…” (P20, PrEP user, 30 years). These online experiences may be seen as joining a PrEP community of GBM.

### 3.9 Adherence to PrEP

PrEP users described various strategies for taking their medication, often borne of trial‐and‐error; many reported having never missed a dose, some occasional missed doses: “I missed the odd dose for like the night‐time ones” (P23, PrEP user, 38 years). Some participants described online groups and blogs as sources of support in navigating adherence.

Non‐PrEP users generally anticipated that adherence would not be a concern: “I do vitamins every day. I can just add that to my regimen…” (P5, non‐user, 47 years). Alternately, a few identified adherence concerns as a deterrent:

I know it's just one pill a day, but when I had to start taking [another drug] and I only took it once a day, I would forget sometimes. And, it made me start to think, oh my god, what if I was on HIV meds or PrEP… (P4, non‐user, 24 years).

When asked about their perspectives on adherence to a once‐daily tablet compared to on‐demand PrEP (not yet approved by Health Canada, but prescribed off label), some participants described a preference for the stability of a once‐daily tablet given the spontaneous and erratic nature of their sex lives; others identified a lack of professional guidance about how to use an on‐demand regimen.

### 3.10 Retention/discontinuation

Among fifteen participants who received prescriptions for PrEP, eleven (73.3%) were retained in PrEP care: one participant never started taking it and three reported stopping PrEP use – two intermittently (four to six weeks) and one completely. Those who ceased taking PrEP described weighing the risks and benefits, including financial costs during periods of low levels of sexual activity:

I don't know what it adds up to [per pill], but I was really sick for five or six weeks. I just stopped taking it because I'm not going to hook up so I'm just going to stop, and then I started again when I started feeling better (P26, PrEP user, 34 years).

Several participants described their anticipated PrEP use timeframe as “indefinitely” (P24, PrEP user, 31 years), “until I die” (P7, PrEP user, 45 years) and “there is no way I can go off it” (P23, PrEP user, 38 years). Others perceived using PrEP for a limited time, reporting their PrEP use may change due to relationship status (e.g. entering a monogamous relationship) or insurance coverage (e.g. losing a job).

PrEP users largely did not report concerns, and several indicated benefits, around ongoing HIV testing and engagement with care: “I think getting tested regularly as a community is one of the best ways to reduce new STI transmission” (P20, PrEP user, 30 years). When asked about possible alternatives, both PrEP users and non‐users expressed interest in injectable PrEP, perceived as offering protection without daily adherence and eliminating the psychological burden of everyday antiretroviral use: “I think that would be cool, and then you could just forget about it, because I think it could be psychological for some people” (P28, non‐user, 28 years). All participants hoped for long‐term protection from an efficacious HIV vaccine, described as “ideal.”

### 3.11 PrEP stigma

Participant narratives revealed acute awareness of stigma associated with PrEP use (and users) and GBM's sexuality more broadly, evidenced among PrEP users and non‐users. Vicarious stigma was revealed in assumptions about PrEP users, including reading and hearing stories, especially online: “The internet and the apps are opportunities to be really cruel for many people” (P1, non‐user, 33 years). A PrEP user similarly described the Internet as a vehicle for stigma, but also challenging such judgements:

He [a friend online] was very judgmental. His post was ‘people [on PrEP] are disgusting.’ I said, listen, I bareback and I don't think I'm a disgusting, horrible person; so, I don't understand why this is an issue when you consider me to be your friend (P7, PrEP user, 45 years).

Negative representations of PrEP users constrained some participants from disclosing their PrEP use due to fears of being judged as “promiscuous” or “barebackers” (felt‐normative stigma); this evoked ambivalence about engaging with other PrEP users in online forums, otherwise noted as a source of support. Negative representations of PrEP users dissuaded some non‐users from discussing or accessing PrEP.

Internalized stigma was revealed in participants’ narratives of ambivalence and shame about using PrEP: “I do feel embarrassed to tell that I'm on PrEP” (P18, PrEP user, 31 years). Non‐users also recounted internalized stigma reflecting negative societal attitudes towards GBM's sexuality more broadly.

PrEP users also reported enacted stigma: “When I talk with gay men, it usually starts off with, ‘You're a slut; is that why you're on it?’” (P10, PrEP user, 27 years). Non‐users similarly reported enacted stigma when engaging in conversations about their consideration of PrEP use. Multiple forms of stigma impacted on PrEP non‐users and users across the PrEP cascade.

### 3.12 Impact of PrEP on sexual practices and relationships

Participant narratives invoked the broader impact of PrEP, beyond the cascade, on sexual decision‐making, negotiations and relationships. A core representation of PrEP was the equation of empowerment and choice with condomless sex: “Yeah, I guess that's the beauty of PrEP; it empowers us to actually state what kind of sex we want. Without that condom, condom, condom, condom” (P12, PrEP user, 42 years). Another participant described his evolution along this continuum:

I was in disbelief for a really long time, that it was actually going to work. I was of the mind that, well, I'll take it, but I'll still continue to use condoms in specific situations. But that faded really quickly and I started having a lot of bareback sex. It became my default (P11, PrEP user, 33 years)

Both PrEP‐experienced and PrEP‐naïve participants described challenges in navigating sexual relationships in the context of PrEP. From a PrEP user's perspective, “Now if I'm talking to two people, one of them says condom and the other one doesn't, it's 99% that I'll go with the one who says no condoms” (P27, PrEP user, 37 years). Non‐users described experiences of being encouraged not to use condoms, often ending with rejection from PrEP users:

…we were about to close the deal, and then the disclosure would come from these folks that they were on PrEP…would I like to have sex with them without a condom? Immediately it was totally cut‐off, and it threw me a little bit (P1, non‐user, 33 years).

Another narrative emerged around STIs, with some PrEP users describing relative lack of concern about STIs in contrast to HIV infection: “The concern was that I'd get more STIs. I did, but it still wasn't worth not being on PrEP” (P23, PrEP user, 38 years).”

## 4 Discussion

This in‐depth exploration of GBM's perspectives and experiences in encountering and negotiating PrEP use – in the clinic and in the community – invokes elements of a PrEP cascade, with challenges and opportunities at various stages; however, it also reveals substantial gaps in the cascade. The application of a linear continuum of care to understand PrEP use/non‐use overlooks alternate trajectories and goals for PrEP use, and may deemphasize psychological/affective, interpersonal, social and community phenomena that impact on PrEP implementation and the broader psychological and sexual health of GBM.

Our findings reveal challenges to the conceptualization of a PrEP user/non‐user binary, as well as PrEP use trajectories that do not map onto a linear cascade or culminate in a goal of multi‐year use and retention in care 8, 19, 30. PrEP users and non‐users largely expressed similar sexual practices, but for the most part only PrEP users perceived themselves as engaging in high‐risk sexual practices that warranted PrEP use. Low perception of HIV risk among those considered objectively at high risk has been described as a principal barrier to PrEP uptake 42. However, we identified other motivations for PrEP use, including the opportunity to safely decrease condom use in order to enhance sexual pleasure and that of reducing the widespread psychological burden of HIV‐related anxiety. These motivations underscore participants’ focus on sexual and psychological wellbeing, in addition to physical wellbeing (i.e. reduced HIV vulnerability), corroborating other emerging research with PrEP users 16, 43, 44, 45.

PrEP “non‐users” in our study were situated at various positions, from staunchly against PrEP use to considering PrEP in the present or for the future. Although this may appear to mirror the early stages of change in the transtheoretical model 46, which has been applied to understand PrEP uptake 14, it is vital for practitioners and researchers to understand and respect that PrEP uptake is not a desired or viable option for all GBM, regardless of sexual practices.

Further complicating the “cascade,” among GBM considered “PrEP users,” one decided to access PrEP but did not fill his prescription; others initiated PrEP but then discontinued; and, of these, some re‐initiated PrEP and others did not. Becoming or staying adherent were not universal goals; rather, participants revealed ongoing decision‐making about whether to take and remain on PrEP based on dynamic sexual risk and relationship trajectories. Many participants saw themselves as being on PrEP for a finite amount of time, indicating the shortcomings of analogizing PrEP for HIV‐negative people to antiretroviral medication for people living with HIV and an HIV continuum of care. “Retention in PrEP care” appears not to be an accurate representation of the end stage (or “success”) of the PrEP cascade; rather, PrEP discontinuation and various permutations of seasonal or intermittent use 8, 47 were also desired goals.

Among the potent psychosocial phenomena revealed was the impact of PrEP in freeing GBM from decades‐long anxiety about HIV that had pervaded their sexual and social words 19, 31, 32, 43, 48; this may be difficult to capture in quantitative metrics designed to assess progress along the PrEP cascade. Nevertheless, this affective dimension is likely to influence GBM's encounters with PrEP, from awareness, to decision‐making about initiation, to adherence and retention.

A second psychosocial dimension was acute awareness of PrEP stigma and its impact across the cascade 26, 27, 30, 32, 44. To the extent stigma is not depicted as a social phenomenon throughout the cascade, this may result in missed opportunities to mitigate foreseeable obstacles to PrEP access, initiation and adherence; it also may exacerbate individual GBM's internalization of stigma (due to its being constructed as an individual rather than a social‐structural problem), with negative impacts on psychological and sexual health.

Third, we identified the potential for shifts in sexual practices that present new challenges for risk reduction approaches, including sexual negotiation and seroadaptive strategies 49, which have become GBM community norms for two decades. We did not identify extensive changes in the sexual practices of individual GBM post‐PrEP initiation, similar to other investigations 19, 31, 35, 44 – most participants were already using condoms inconsistently. However, a number of emerging tensions were evoked in GBM's sexual practices and negotiation. These included experiences of increased pressure to engage in condomless anal sex – among both PrEP users and non‐users, increased challenges in negotiating condom use, and perceived increases in rejection by potential sexual partners of those GBM who insisted on using condoms.

Future research involving PrEP‐experienced and PrEP‐naïve GBM is necessary to understand how PrEP may be best represented and implemented in the service of empowering GBM, both those who choose to adopt PrEP 45, 48, 50 and those who do not, with the aim of promoting sexual agency and community health. Our findings affirm those of recent qualitative studies with PrEP‐naïve GBM and HIV‐positive GBM that illustrate how PrEP may foment fault‐lines within GBM communities along an HIV‐positive/HIV‐negative sero‐divide, and the shortcomings of technologically mediated approaches to HIV prevention that fail to meaningfully engage with GBM's lived experience 24, 51; in the present study with GBM PrEP users and non‐users, we identified inchoate schisms along a PrEP user/non‐user divide. Significantly, a unilateral biomedical approach to PrEP (e.g. “getting drugs into bodies” 21) risks equating empowerment and sexual health solely with PrEP uptake – rather than with making one's own informed choices – and thereby may alienate those GBM who do not find PrEP a desirable option from public health and HIV preventive interventions, and GBM from each other.

**Figure 1:**
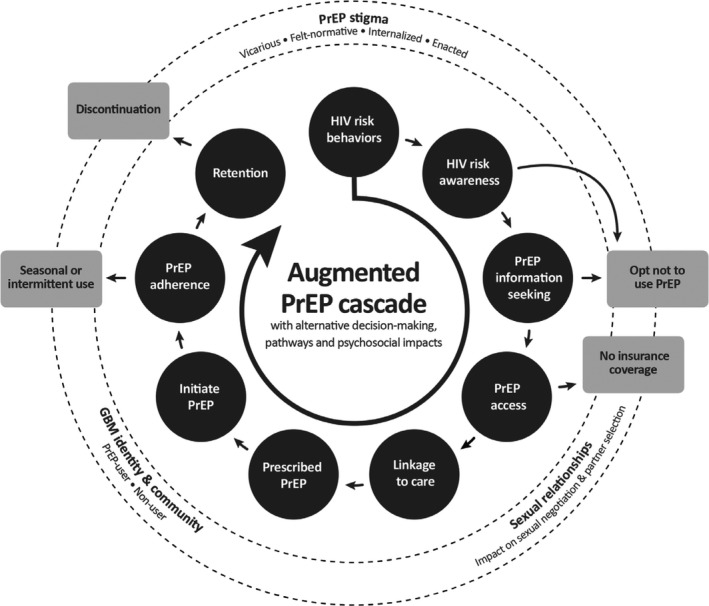
An augmented PrEP cascade incorporating alternate decision‐making and endpoints, and psychosocial challenges, among gay, bisexual and other men who have sex with men PrEP, pre‐exposure prophylaxis.

Based on our findings, we propose an expanded PrEP cascade which incorporates (1) multiple/alternative trajectories of PrEP use, and (2) psychosocial, interpersonal and community‐level challenges that may be anticipated as PrEP is introduced and rolled out (see Figure 1). Explicit acknowledgement of foreseeable psychosocial and community challenges among GBM in considering and encountering PrEP may contribute to mitigating PrEP stigma; to supporting re‐tooled harm reduction strategies; and to facilitating communication at interpersonal and community levels, with opportunities for targeted programmes and policies to support GBM's sexual and psychosocial health – not merely PrEP use.

### 4.1 Strengths and limitations

This is among the first qualitative studies to include both PrEP‐experienced and PrEP‐naïve community‐recruited GBM in exploring experiences and psychosocial considerations affecting PrEP decision‐making and sexual relationships in the PrEP era. The relatively small sample of GBM recruited in one urban centre, known to be welcoming of sexual minorities, suggests caution in generalizing the findings to GBM in smaller or less accepting communities; however, they may be transferable to urban GBM in other high‐income countries. This study was begun pre‐licensure of PrEP in Canada. Contrary to a US‐based qualitative study which identified some participants' hesitancy to use PrEP pre‐FDA approval^25^, this did not emerge in our study, perhaps due to the earlier US approval. However, lack of licensure may have presented barriers to uptake due to the unavailability of insurance coverage.

## 5 Conclusions

This study suggests the need for an expanded “PrEP cascade” that addresses dynamic behavioural risks which may benefit from seasonal or intermittent use, systemic barriers in access to and sustaining PrEP use and an end goal of terminating PrEP. An integrated PrEP cascade that incorporates GBM's lived experiences, evolving preferences, and psychosocial and community‐level challenges, in alignment with a combination prevention model, may more effectively promote PrEP implementation, and GBM's broader sexual and psychological health.

## Competing interests

The authors have no declarations or conflicts of interest associated with this work.

## Authors’ contributions

PAN and AG designed the study and data collection tools, and participated in data analysis. AG conducted the data collection. ALD and ST analysed the data. ALD drafted the initial manuscript. PAN revised the manuscript. All authors contributed to drafting and finalizing the manuscript.

## Appendix 1

### 1.1

**Appendix 1. Key themes, dimensions and exemplar quotations from gay, bisexual and other men who have sex with men in Toronto, Canada (N = 29), October 2015 to March 2016**

**Table:**

Themes and dimensions	Exemplar quotations
1. HIV risk behaviours	
Inconsistent condom use/dislike condoms	“…For me, condoms sometimes either broke and made me really upset and worried. Some other times, I have to admit, I didn't like them, and so it was 50‐50, it was not consistent, and it really worried me.” (P18, PrEP user, 31 years)
Inconsistent condom use/prefer non‐use	“Well, it was I would say 50‐50 depending on how I felt, the level of comfort that I had, or the other person may have certain concerns. Yeah, it was about 50‐50, condoms versus no condoms.” (P7, PrEP user, 45 years)
Consistent condom use	“Well, I also don't have a baseline for condomless intercourse, I didn't, so I didn't have any feeling of missing out on anything, or what have you, so condom usage for me as a top wasn't a problem, and condom usage in a relationship to me as a bottom, well, that was simply mandatory. If you don't want to use a condom with me, I'm sorry, I'm not going to be bottoming for you bareback.” (P17, Non‐user, 36 years)
2. HIV risk awareness	
HIV anxiety	“I have had anxiety for a couple of years now, and part of that is I would constantly worry about getting cancer, getting HIV. So, even times when I was safe, there would be something in the back of my mind like, ‘what if?’” (P22, PrEP user, 23 years)
HIV anxiety/ambivalence	“So, as soon as something happens that's risky and, quite frankly, feels good, the next thought is well, I've got a cough, I've got a cold. Am I positive?” (P10, PrEP user, 27 years)
STI risk concern	“…Yeah, I could potentially have bareback sex, but PrEP isn't going to protect me from all the other STIs out there, and now I'm learning that some of them are much more easily transmitted than HIV. So, that's a challenging point for me.” (P4, non‐user, 24 years)
Fear of risk compensation	“To be honest it was like, really, we are going to start taking a pill now to prevent ourselves from getting HIV? And I saw it as almost like a Russian roulette approach to having unprotected, risky sex….” (P13, non‐user, 38 years)
3. PrEP information seeking	
Engage with information/consider for oneself	“So, I read about it for a while but I really only started thinking about it last year, relevantly, if it might be relevant to me.” (P28, non‐user, 28 years)
Process of increasing knowledge/understanding	“It's an ongoing process. It's not just, I woke up one morning, I think I'm going to take PrEP now. It's more of a, over the years, I've learned more about PrEP through research, talked to a lot of people, talked to people who work in this field, who are using it as well.” (P12, PrEP user, 42 years)
Not considering PrEP	“If I was considering it, I'd probably do some more serious research on it….” (P5, non‐user, 47 years)
4. PrEP access	
Inaccessible/high cost	“… Well, the cost would have been such that it would have been inaccessible to me so I really didn't consider it on any kind of basis” (P17, non‐user, 36 years)
Covered by insurance	“And, thankfully, I have coverage through work. I was able to get my medication covered. It's over $1,000 a month, so it really helps” (P7, PrEP user, 45 years)
Uncertain coverage due to job/insurance loss	“Now that I'm in the process of losing my job and my insurance benefits and finding out new benefits and all that kind of stuff, it's really quite interesting that that administrative process forces you into this whole, holy crap, what could happen to my health in the next year?” (P10, PrEP user, 27 years)
5. Linkage to PrEP care	
No perceived obstacles/gay doctor	“He's a gay doctor. I've talked to him about lots of stuff. I would have no problem asking him. He'd probably just, oh really, okay. What's going on?” (P5, non‐user, 47 years)
Preference for gay doctor	“I mean doctors are not supposed to judge you obviously, but at the same time I would like to talk to someone who knows what I'm going through. I don't have to explain certain things to him before he can give me his professional opinion. No, it's great. I tell people when they ask me about PrEP, I tell them, do you have a gay doctor, you really need to find a gay doctor. I'm sure yours is great, but only a gay doctor would really understand, once again in my opinion.” (P7, PrEP user, 45 years)
6. Prescribed PrEP	
Positive experience with healthcare provider	“It was kind of a cool experience, I guess, just leaving there like…” (P10, PrEP user, 30 years)
Streamlined process	“…when I eventually did go in, it was really straightforward… conversation about why I wanted to take it, what I knew about it, and then do the blood work and that kind of stuff. It was easily within a month of bringing it up, to getting started” (P20, PrEP user, 30 years)
Positive experience with pharmacist	“She was actually very cool about it…Her pharmacy is right in the gay village.” (P19, PrEP user, 48 years) “The pharmacist that I see is well‐versed in PrEP…and the [pharmacy] deals specifically in HIV medication every day….” (P10, PrEP user, 27 years).
7. Initiating PrEP	
Sense of accomplishment	“So many months had led up to it and it was like finally I've taken my first step. I first heard about this in late 2014 and early 2015, and now it's getting close to late 2015.” (P22, PrEP user, 23 years)
Happiness	“[I did the] first pill selfie and 90‐day check‐in, yeah…I felt pretty happy.” (P10, PrEP user, 30 years)
“Non‐event”	“[it was] a complete non‐event, it was just a pill.” (P14, PrEP user, 42 years)
Rumination	“I went through…I sat down in my room, and I thought…I remember thinking if it was the right thing to do.” (P18, PrEP user, 31 years)
8. Adherence to PrEP	
Missed dose	“Sometimes the day gets away from you, and I would forget.” (P24, PrEP user, 31 years)
Intermittent use as challenging	“But, the problem is there seems to be no clear consensus as to how to take it if you're doing that [taking it intermittently]. So, I'd rather just not risk it.” (P22, PrEP user, 23 years)
Strategies for adherence	“…I made a schedule. I made an alarm. I made a plan that I have to take my pills before 10:00 with my alarm. So, that makes a very annoying sound. And it doesn't matter if I would be at work or wherever. I have to cut everything and take my pill and then turn it off.” (P3, PrEP user, 39 years)
9. Retention/discontinuation	
Benefits of HIV testing	“That's another thing too is that I'm guaranteed to go get tested for everything every three months, whereas before maybe every six months it was happening or so. So, it forces me to get tested, which if I do have anything, I can kill it pretty quick.” (P22, PrEP user, 23 years)
Time‐limited use	“Yeah I told my partner, I said I really didn't want to take it for more than a year continuously. I really don't want to.” (P19, PrEP user, 48 years)
Routinization of care	“Every three months, yeah, I get them pre‐printed in advance. He gives me all my lab requisitions, so I just go in and do them the week before and then I go in and get swabs and all of that.” (P10, PrEP user, 27 years)
10. PrEP stigma	
Felt‐normative stigma	“There was one big conversation I had with a friend about a mutual friend, who we found out publicly was on PrEP, and both of us were like, ‘what?’ The first thing is, ‘well, of course, they're on it. You know what they're into, right?’” (P2, non‐user, 38 years)
Vicarious stigma	“The internet and the apps are opportunities to be really cruel for many people, and I have seen language used around, ‘oh, silly faggot taking loads, get some self‐respect’, those sorts of things.” (P1, non‐user, 33 years)
Enacted stigma	“He [a friend online] was very judgmental in terms of coming down on people who choose to bareback and are taking PrEP as a preventative measure. His post was ‘people [on PrEP] are disgusting,’ this and that. I said, listen, I bareback and I don't think I'm a disgusting, horrible person, so I don't understand why this is an issue when you consider me to be your friend.” (P7, PrEP user, 45 years)
Internalized stigma	“Knowing myself, if I was to take PrEP my behaviour would change. A friend that isn't on it, said I would just be the biggest cum slut out there if that was the case.” (P5, non‐user, 47 years) “I don't put it on my profile. But, I tell people if I think it helps.” (P22, PrEP user, 23 years) “I do feel embarrassed to tell that I'm on PrEP. Even with the person that I'm seeing now… So I guess there is a little bit of shame in me.” (P18, PrEP user, 31 years)
11. Impact of PrEP on sexual practices and relationships	
Pressure not to use condoms	“…I'd say it was like a year ago or so the pressure to have sex bare, it's like overnight and like through the roof.” (P14, PrEP user, 42 years)
Condomless sex as norm	“Right, because I really haven't met anybody yet that takes PrEP and hasn't asked me to do bareback sex. Usually it comes up, do we need to use condoms?” (P8, non‐user, 50 years)
Rejection due to insisting on condom use	“…we were about to close the deal, and then the disclosure would come from these folks that they were on PrEP…would I like to have sex with them without a condom? I said well, the guideline that I have with my man is that we use condoms when we're having sex outside our relationship. Immediately it was totally cut‐off, and it threw me a little bit.” (P1, non‐user, 33 years)
STIs as relatively inconsequential	“… I think the other STDs like gonorrhea, chlamydia, like syphilis, they're in a way curable… You get something, you get an antibiotic, you go home and you survive.” (P26, PrEP user, 34 years)

PrEP, pre‐exposure prophylaxis; STDs, sexually transmitted diseases; STIs, sexually transmitted infections.
